# Hydrogen Peroxide Signaling in Physiology and Pathology

**DOI:** 10.3390/antiox12030661

**Published:** 2023-03-07

**Authors:** Christine Rampon, Sophie Vriz

**Affiliations:** 1 Laboratoire des BioMolécules (LBM), Département de Chimie, École Normale Supérieure, PSL University, Centre National de la Recherche Scientifique (CNRS), 75005 Paris, France; 2 Sorbonne Université, 75006 Paris, France; 3 Faculty of Sciences, Université Paris-Cité, 75206 Paris, France

Reactive oxygen species (ROS) were originally described as toxic by-products of aerobic cellular energy metabolism associated with the development of several diseases, such as cancer, neurodegenerative diseases, and diabetes [1,2,3]. In these contexts, the accumulation of ROS in cells, referred to as oxidative stress, is a toxic event that damages a number of biomolecules. Over the last thirty years, research has focused on the development of strategies to reduce ROS in order to prevent tissue damage in normal aging tissues and in pathological situations. An industry related to “antioxidant” strategies expanded to the mainstream, and nowadays everybody seems aware of the toxicity of ROS.

However, recent findings have shown that ROS can also contribute to bona fide physiological processes, leading to a new paradigm in reversible post-translational modifications involved in signal transduction, defined as oxidative eustress [4,5]. Amongst ROS, hydrogen peroxide (H_2_O_2_) best fits the properties of a signaling molecule and is recognizable as the major ROS in the oxidative regulation of physiological activity [6]. H_2_O_2_ is mainly produced by NAPDH oxidases and the mitochondrial electron transport chain [7,8]. This generation is controlled by growth factors, chemokines and physical stress, among other factors.

This Special Issue highlights the most recent advances in all the aspects of ROS signaling with examples of H_2_O_2_ signaling in *E. coli*, plants and animals. It has recently been shown that H_2_O_2_ can regulate Shh signaling during development and regeneration [9]. Thauvin et al. analyzed its molecular mechanism and discovered that Shh controlled H_2_O_2_ levels through a noncanonical Boc-Rac1 pathway [10]. In a positive loop, H_2_O_2_ regulates Shh trafficking. Thus, Shh directly impacts its own distribution and potentially the distribution of other morphogens via H_2_O_2_ level modulation. These founding results provide a molecular explanation for the robustness of morphogenesis and open a new path toward the integration of ROS regulation in morphogens signaling. It has been shown that ROS level oscillation is involved in cell cycle regulation in vertebrate early development [11]. Tokmakov et al. showed that ROS levels are also involved in fertilization via the control of calcium in *Xenopus laevis* oocytes [12]. This calcium and H_2_O_2_ signaling crosstalk is also at work in plants, as shown by Cheng et al. in this Issue [13]. In this article, the authors investigate the molecular targets of H_2_O_2_ and Ca^2+^ in melon and *Arabidopsis* seed germination. They show that H_2_O_2_ and Ca^2+^ form a reciprocal positive-regulatory loop to maintain a balance between abscisic acid (ABA) and gibberellic acid (GA_3_) essential to promoting seed germination under ABA stress. Finally, Roth et al. performed a transcriptomic analysis of *E. coli* response to different concentrations of H_2_O_2_ [14]. This analysis reveals that different stress responses are activated by H_2_O_2_ exposure and emphasize the role of cysteine synthesis as an antioxidant response. 

We would like to acknowledge the authors that have contributed to this Special Issue, “Hydrogen peroxide signaling in physiology and pathology”. This Special Issue has highlighted the need for further research into the mechanisms of ROS signaling.
